# Editorial: Advancements in immunology and immunotherapy for breast cancer

**DOI:** 10.3389/fonc.2022.979806

**Published:** 2022-08-09

**Authors:** Tomoharu Sugie, Roberto Salgado, Lawrence Fong

**Affiliations:** ^1^ Breast Surgery, Kansai Medical University Hospital, Hirakata, Japan; ^2^ Department of Pathology, GZA-ZNA-Hospitals, Antwerp, Belgium; ^3^ Division of Research, Peter Mac Callum Cancer Centre, Melbourne, VIC, Australia; ^4^ Division of Hematology/Oncology, University of California, San Francisco, San Francisco, CA, United States

**Keywords:** Breast cancer, Immunotherapy, Tumor microenvironment, Programmed death ligand (PD-L) 1, Biomarker

Breast cancer is a common disease in women in worldwide. Although conventional approaches such as surgery, radiotherapy chemotherapy, and endocrine therapy contribute to the submission of early breast cancer, they are limited by specificity and toxicity in advanced metastatic breast cancer. Accumulated results regarding immunogenicity and immune response in breast cancer have led to the development of immunotherapy for patients with early-stage as well as advanced breast cancer (1). As Chen et al. summarized, the therapeutic effects of immuno-oncology (IO) for breast cancer have been observed but are still limited. Among the subtypes of breast cancer, triple-negative breast cancer (TNBC) has the most aggressive features and the conventional treatment has been limited to chemotherapy. As Ji et al. have described, immunotherapies for breast cancer are focused on TNBC with the combined immunotherapy, including immune checkpoint inhibitors (ICIs), plus chemotherapeutic drugs, molecular target drugs, or radiation. However, there is still room for improvement as new strategies for IO are in progress as mentioned by Qiu et al. The combined agents might have direct cell toxicity, release immunomodulatory factors from dying tumor cells, induce the infiltration of immune cells, and suppress immune regulatory cells. Optimal combinations and sequences of immune-based therapies should be determined.

Programmed death-ligand 1/programmed cell death-1 (PD-L1/PD-1) represent a valuable therapeutic target, but Cong et al. reported that T-cell immunoglobulin mucin-3 (TIM3), another immune checkpoint molecule, is a potential target for IO. The PD-L1 test is the mainstream for companion diagnostic of ICIs, but the development of biomarkers is required to predict prognosis and/or responses to IO to maximize the clinical benefit of ICIs. Shang et al. examined tumor infiltrating lymphocyte (TIL) and PD-L1 expression in relation to effectives of HER2-targeted therapy. High TIL infiltration before NAC was a strong predictive marker for pathological complete response (pCR) which was consistent with the results of the previous study (2). PD-L1 expression on tumor cells is regulated in the tumor-intrinsic and -extrinsic manner. PD-L1 expression before NAC represents a naïve anti-tumor response while PD-L1 after NAC is the result of the immune contexture in tumors treated with anti-cancer agents. The novel technologies of cancer research are in the advance stages and Magbanua et al. summarized liquid biopsy for circulating tumor DNA (ctDNA) analysis. For urothelial carcinoma, ctDNA proved to be a promising biomarker to predict clinical outcomes in patients with adjuvant atezolizumab (3) and this dynamic molecular biomarker was warranted.

For immuno-related biomarkers, Chang et al. have examined immunoglobulin lambda constant 2 (IGLC2) in TNBC and the low gene expression of IGLC2 was correlated with a poor prognosis and malignant features of TNBC. IGLC2 may contribute to inflamed gene expression profiles but it is still obscure as to why this humoral immune marker is related to cellular anti-tumor immunity. Accumulated results indicated that the presence of B cells and tertiary lymphoid structure (TLS) in tumors were associated with a favorable outcome in patients treated with immunotherapy and this relationship between humoral and cell-mediated immunity gradually became clear (4). Innate immunity – related molecule of CRAR2, the second receptor of complement 5a (C5a), was also a potential biomarker for immune response. Zhu et al. reported that C5AR2 expression was a poorer prognostic factor in breast cancer, especially the ER-positive subtype. Monocyte-macrophages are heterogenous and divided into two subtypes for anti-tumor M1 and pro-tumor M2 macrophages. Tumor-associated macrophages (TAMs), which created an immune-suppressive environment, tended to express the markers for M2 macrophage. Expression levels of C5AR2 were positively correlated with the infiltration of M2 macrophages but negatively correlated with the infiltration of M1macrophages. Liu et al. reported that the high gene expression of GOLT1B, encoding a golgi vesicle transporter protein, was a negative prognostic factor. This gene may contribute to the infiltration of immune cells. The expression level of GOLT1B was negatively correlated with CD8-positive effector T cells, CD4-positive helper T cells, regulatory T cells, and positively correlated with M2 macrophages and neutrophils. These results indicated that both C5rAR2 and GOLT1B were a potential negative predictive biomarker in relation to pro-tumor immunity. Xu et al. identified GW-8510, a CDK2 inhibitor, as an anti-tumor response enhancer using the bioinformatics manner. TNBC cancer cell treatment with GW-8510 increased the level of cleaved caspase-3 and N-terminal fragments of GSDME, which induced pyroptosis, a lytic programmed cell death. Pyroptosis cells released damaged associated molecular patterns (DAMPs), which augmented an anti-tumor response in tumor microenvironment (TME). Neoantigen sources were mainly on single nucleotide variants (SNVs) and small insertion-deletion(indel), which are a potential target for immunotherapy (5). The FDA has already approved pembrolizumab for metastatic solid tumors with tumor mutation burden-high (‗ 10 mut/Mb) or microsatellite instability-high. Zhou et al. reported that PIK3CA is a highly mutated gene and the highest source of neoantigens. Breast cancer in the elderly or breast cancer with ER-positive, HER2-negative yield higher SNV-derived neoantigens. Recent results from Chandran, et al. also demonstrated that mutant PIK3CA-derived public neoantigens had immunogenicity and therapeutic potential (6).

The understanding of molecular and cellular dynamism in TME is required for the development of IO biomarkers. Patysheva et al. highlighted the relation between the response to neoadjuvant chemotherapy (NAC) and circulating monocyte-phenotypes. NAC recruited CD163-positive monocyte-derived macrophages in TME and the circulating CD14pos/lowCD16-positive HLA-DR-positive monocyte in the base-line was associated with NAC efficacy. The accumulation of CD163-positive TAM may result from active recruitment by anti-cancer agents or an adaptive response to inflammation reaction induced by NAC. TIL (CD8-positive T cell) - infiltration which is a favorable prognostic immune marker for breast cancer, especially in TNBC (7, 8). Zhou et al. examined the infiltration of immune cells in three matched samples for normal, primary, and oligometastatic sites. Among matched tissues, immune cell infiltration was less in oligometastatic sites compared to primary sites. Higher CD3 in the intratumor oligometastatic lesion was correlated with better PFS and higher CD4 in the same lesion and was related to better OS in TNBC/HER2-postive breast cancer. CD4-positive T cells modulate cellular (Th1) and humoral (Th2) immunity but intratumor CD4-positive T cells can mediate anti-tumor cytotoxicity in a direct and indirect manner (9). The therapeutic effects of IO depend on how the immune suppressive status of TME is overcome. As regulatory T cells (Tregs) play a major role in the immunosuppression of TME, the targeting of Treg is a promising approach to augment the anti-tumor response (10). Liu et al. summarized Treg-biology and the rationale for Treg-targeting treatment in breast cancer.

Vitorino et al. summarized gut microbiota which influences immunotherapy response. Melanoma patients with “good microbiota” experienced the benefit from IO while the fecal transplantation of good microbiota could overcome resistance to IO (11). Pathogen-associated molecular patterns (PAMPs) from microbiota may activate local gut-innate immunity but it is still unclear why they can induce systemic CD8 T cell-based anti-tumor immunity. Further study is required to elucidate the contribution of gut microbiota as related to systemic anti-tumor immunity.

In conclusion, anti-PD-(L)1 is now FDA approved for TNBC, both in the neoadjuvant and metastatic setting, in combination with chemotherapy. We do not really know why chemotherapy should synergize with immunotherapy, but this treatment may have broad effects on TME-mediated immunosuppression. Future work should integrate both tumor-cell intrinsic and extrinsic determinants of responsiveness to immunotherapy. This would enable biomarkers for improved patient selection as well as new resistance mechanisms that could be co-targeted in combination strategies. Liquid biopsies could enable accelerated development of treatments as well as tailor treatments for potential real-time response.

## Author contributions

Original draft preparation: TS. Review and editing: RS and LF. All authors contributed to the article and approved the submitted version.

## Conflict of interest

TS reports research funding from Chugai/Roche, Eisai and Kyoto Bridge for Breakthrough Medicine, honoraria from Chugai/Roche, Merck Sharp & Dohme (MSD), Astra Zeneca, Pfizer, Eli Lilly, Taiho, Daiichi Sankyo and Eisai. RS reports non-financial support from MSD and Bristol Myers Squibb (BMS), research support from MSD, Puma Biotechnology and Roche, and personal fees from Roche, BMS and Exact Sciences for advisory boards. LF reports research funding to the institution from Abbvie, Amgen, Bavarian Nordic, BMS, Dendreon, Janssen, MSD, and Roche/Genentech. LF reports consulting or advisory role with BMS, Daiichi Sankyo, Innovent, MSD, Roche/Genentech, and EMD Serono.

## Publisher’s note

All claims expressed in this article are solely those of the authors and do not necessarily represent those of their affiliated organizations, or those of the publisher, the editors and the reviewers. Any product that may be evaluated in this article, or claim that may be made by its manufacturer, is not guaranteed or endorsed by the publisher.
